# Electroretinographic responses to luminance and cone-isolating white noise stimuli in macaques

**DOI:** 10.3389/fnins.2022.925405

**Published:** 2022-07-29

**Authors:** Jan Kremers, Avinash J. Aher, Neil R. A. Parry, Nimesh B. Patel, Laura J. Frishman

**Affiliations:** ^1^Section for Retinal Physiology, University Hospital Erlangen, Erlangen, Germany; ^2^Vision Science Centre, Manchester Academic Health Science Centre, Manchester Royal Eye Hospital, Manchester University NHS Foundation Trust, Manchester, United Kingdom; ^3^Faculty of Biology, Medicine and Health, University of Manchester, Manchester, United Kingdom; ^4^Department of Vision Sciences, College of Optometry, University of Houston, Houston, TX, United States

**Keywords:** electroretinogram, L-cones, M-cones, macaque, luminance, temporal white noise stimulation, impulse response functions, modulation transfer

## Abstract

Electroretinograms (ERGs) are mass potentials with a retinal origin that can be measured non-invasively. They can provide information about the physiology of the retina. Often, ERGs are measured to flashes that are highly unnatural stimuli. To obtain more information about the physiology of the retina, we measured ERGs with temporal white noise (TWN) stimuli that are more natural and keep the retina in a normal range of operation. The stimuli can be combined with the silent substitution stimulation technique with which the responses of single photoreceptor types can be isolated. We characterized electroretinogram (ERG) responses driven by luminance activity or by the L- or the M-cones. The ERGs were measured from five anesthetized macaques (two females) to luminance, to L-cone isolating and to M-cone isolating stimuli in which luminance or cone excitation were modulated with a TWN profile. The responses from different recordings were correlated with each other to study reproducibility and inter-individual variability. Impulse response functions (IRFs) were derived by cross-correlating the response with the stimulus. Modulation transfer functions (MTFs) were the IRFs in the frequency domain. The responses to luminance and L-cone isolating stimuli showed the largest reproducibility. The M-cone driven responses showed the smallest inter-individual variability. The IRFs and MTFs showed early (high frequency) components that were dominated by L-cone driven signals. A late component was equally driven by L- and M-cone activity. The IRFs showed characteristic similarities and differences relative to flash ERGs. The responses to TWN stimuli can be used to characterize the involvement of retinal cells and pathways to the ERG response. It can also be used to identify linear and non-linear processes.

## Introduction

Electroretinograms (ERGs) are typically recorded in response to light flashes. The flash ERG is still very often used to assess retinal physiology in the clinic and in research. Stimuli, recording procedures and analyses for the full field flash ERG have been standardized by the International Society for Clinical Electrophysiology of Vision (ISCEV) to enable comparisons of the results obtained in different centers and studies. However, flashes have the disadvantage that a large amount of energy is compressed in a short presentation time. The ISCEV standards recommend measurements with flashes as high as 10 cd.s/m^2^ under dark-adapted conditions. ISCEV also recommends that the flash times do not exceed 5 ms (Robson et al., 2022). Assuming that the pupil of the subject is dilated to 8 mm diameter, the 5 ms 10 cd.s/m^2^ flash will result in a brief retinal illuminance of approximately 100,000 td. Shorter times of flashes with the same strength will result in even higher retinal illuminances. The standard light-adapted flash is 3 cd.s/m^2^. For either a light- or dark-adapted retina, these are most probably stimuli with little ecological relevance, even when it may have a clinical application in diagnosing and monitoring retinal disorders and may give valuable information about photoreceptor physiology.

With the advent of different techniques to create visual stimuli, such as monitors or light emitting diodes (LEDs), other stimulus types can be created relatively easily. Responses to these stimuli may be able to give additional information about retinal signal processing. For instance, with monitors, multifocal stimulation became possible and the spatial distribution of the flash ERG could be obtained (Sutter and Tran, 1992; Hood, 2000). LEDs have excellent temporal resolution and, in principle, a large variety of temporal stimuli can be created, including continuous waveforms such as sine waves (Viswanathan et al., 2002; Kremers et al., 2010, 2021b; Kremers and Pangeni, 2012; Kommanapalli et al., 2014), square waves (McKeefry et al., 2014) and sawtooth waveforms (Kremers et al., 2014; Tsai et al., 2016). In addition, LEDs can have many different small band emission spectra (i.e. colors) enabling the creation of many different chromaticities and therefore the control of the activity in different photoreceptor types and in post-receptoral mechanisms (Kremers, 2003; Kremers and Link, 2008; Challa et al., 2010; Kremers et al., 2010; Kremers and Pangeni, 2012; Kommanapalli et al., 2014; Maguire et al., 2016). These additional stimuli have the potential to give important information about the physiology and pathophysiology of the retina that may go beyond that obtained by the flash ERG.

A possible way to extract useful information about retinal processes in a physiologically relevant range can be obtained from white noise stimulation (Marmarelis and Marmarelis, 1978). White noise stimuli have equal amplitudes across a wide range of temporal frequencies. In the time domain, the stimulus strength (expressed in terms of luminance, chromaticity, photoreceptor excitation etc.) varies in time in a pseudorandom order with a Gaussian output distribution around a mean (see section “Materials and methods”). Unlike flash stimuli, the energy is spread over the whole presentation time and thus can be regarded to be physiologically more relevant. Long recovery times to return to the original state of adaptation, as is required with strong flashes, are not necessary. Furthermore, the state of adaptation is not confounded with changes in stimulus strength (if the time between two flashes is too short to return to steady state as is the case in the ISCEV standard 30 Hz flicker ERG) and thus can be studied as an independent variable. Finally, by using LEDs with different emission spectra, the stimulus can be varied in luminance and chromaticity. When the luminance and chromatic changes are carefully chosen, the responses of single photoreceptor types can be isolated through silent substitutions for the non-desired photoreceptor types (Kremers, 2003). The ERGs elicited by white noise stimuli (wnERGs) can be used to extract impulse response functions (IRFs) by cross-correlating the response with the stimulus in time. In a linear system, the IRF equals the response to an infinitely short flash with infinitely high intensity (and with an energy of 1). Although the use of white noise stimuli in visual electrophysiology is not novel (Marmarelis and Naka, 1973; Field et al., 2010), to our knowledge it has only recently been employed in ERG measurements (Saul and Still, 2016; Zele et al., 2017; Adhikari et al., 2019). In the present study, we explored the utility of temporal white noise stimuli in the macaque monkey. More specifically, we studied the reproducibility of the measured ERGs.

We recently presented the results of ERG measurements in macaque monkeys to repetitive luminance and L- and M-cone isolating stimuli with different temporal profiles (sinusoidal, sawtooth and square wave modulations; Kremers et al., 2021a). The resulting ERGs were compared with those of similar measurements in human subjects. It was found that the responses had similar characteristics in human subjects and in macaques. However, macaque ERG responses were generally more complex and displayed more non-linearities than those obtained in human subjects. It is therefore of interest to perform wnERG recordings in macaques to obtain additional information for direct comparison with other data (e.g., of single neurons) that are obtained from macaques. The purpose of the present study is to investigate the responses to luminance and L- and M-cone isolating white noise stimuli in macaque monkeys. The luminance IRFs can be compared with those obtained previously in human subjects (Zele et al., 2017). Similar as in human subjects, the responses to M-cone driven sinusoidal stimuli were smaller than those to L-cone driven stimuli at high temporal frequencies but of about equal amplitude at low temporal frequencies. We wanted to determine whether L- and M-cone-driven wnERGs and IRFs also differed and how they compare with those driven by luminance stimuli. Finally, the Fourier transform of the IRFs results in a modulation transfer function (MTF) that is comparable with the responses to sine wave stimuli at different temporal frequencies. We therefore compared the MTF with the previously published direct ERG measurements to sine wave stimuli (Kremers et al., 2021a).

For a linear system, the IRFs are expected to be identical to the response to an infinitely strong and an infinitely short flash. The comparison with the flash ERG therefore may provide insights in which components of the flash ERG may be the result of linear or non-linear signal processing in the retina.

Although the ERG may be considered to be an epiphenomenon of visual processing in the retina, it can provide important information about the physiology of retinal neurons and retino-geniculate pathways (Kremers et al., 2020). The wnERGs described in the present paper may provide an efficient and non-invasive method to investigate the properties of these neurons and pathways and can, therefore, be of general interest for studying the visual system and visual perception.

## Materials and methods

### Animals

ERG recordings were performed in five anesthetized adult rhesus monkeys (*Macaca Mulatta*; two females, three males), at the University of Houston (TX, United States). The animals were housed at the University of Houston and had access to food and water *ad libitum*. The recordings presented here were performed with the same animals and during the same recording sessions as those using sinusoidal, square wave and sawtooth stimuli, the results of which were reported previously with details of the preparation procedures (Kremers et al., 2021a). Briefly, macaques were anesthetized by intramuscular injections with ketamine (20–25 mg/kg/h; Ketaved, Vedco, St. Louis, MO, United States), and xylazine (0.8–0.9 mg/kg/h; Ansed, Vedco). They were treated with atropine sulfate (0.04 mg/kg injected subcutaneously; Akorn, St Joseph, MO, United States), as previously described (Rangaswamy et al., 2007; Luo et al., 2014). Body temperature was maintained between 36.5 and 38°C. Heart rate and blood oxygen were monitored. The animals were allowed to breathe freely. After the recordings, the animals were allowed to wake up before they were returned to the housing facilities.

Prior to recording from the right eye, the pupil was fully dilated to approximately 8 mm in diameter with topical tropicamide (1%, Akorn) and phenylephrine (2.5%, Akorn). ERGs were recorded using fiber electrodes (Dawson et al., 1979) placed across the center of the cornea, and covered with carboxy methycellulose sodium and contact lenses to maintain hydration. A session (including the recordings to the other stimuli) lasted between 1 and 2 h.

All experimental and animal care procedures adhered to the ARVO statement for the Use of Animals in Ophthalmic and Vision Research, and were approved by the Institutional Animal Care and Use Committee of the University of Houston (#16-046).

### Stimuli

**FIGURE 1:**
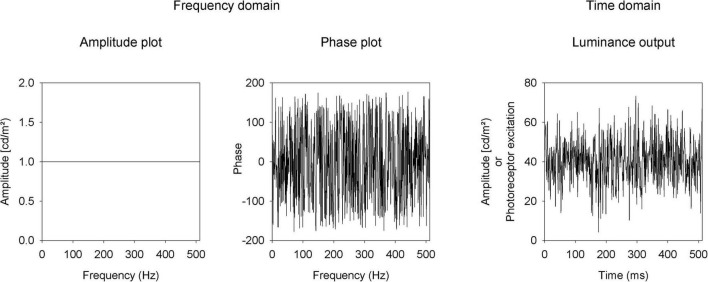
Description of the temporal white noise stimulus as amplitude **(left)** and phase **(middle)** plots in the frequency domain and in the time domain **(right)**.

Luminance and L- and M-cone isolating temporal white noise stimuli (TWN) were created with the Espion E3 system with a ColorDome ganzfeld stimulator (Diagnosys LLC, Lowell, MA, United States). The macaques viewed full field stimuli through a large field stop thereby maintaining what is technically full field stimulation, while excluding the very far periphery (> 100°). In TWN stimuli, the luminance (or cone excitation) as a function of time is pseudorandom and has a Gaussian distribution around the mean luminance. The TWN was created in the frequency domain where all frequencies (F) have identical amplitudes [A(F); Figure 1, left]. Different TWN stimuli differ in the frequency domain by their phase plots (i.e., the phases at different frequencies). To create TWN stimuli, we assigned between 1 and 255 Hz equal amplitudes (luminance of the LEDs in cd/m^2^). The amplitude at 0 Hz was the mean luminance. We assigned random phases [P(F); Figure 1, middle] for these frequencies. We applied an inverse Fourier transform on this stimulus. To be able to perform the inverse Fourier transform, the amplitudes at frequencies 256 + n Hz up to 511 Hz were equal to the amplitudes at 256-n Hz and the phases at 256 + n Hz were equal to those at 256-n Hz multiplied by -1.

Before performing the inverse Fourier transform, the stimulus in the frequency domain was converted into a complex number: C(F) = A(F)* [cos(P(F)) + i*sin(P(F))]. The inverse Fourier transform returned the stimulus (in terms of LED luminances or photoreceptor excitations) in the time domain in 512 equidistant steps of 1 ms (Figure 1, right).

**FIGURE 2:**
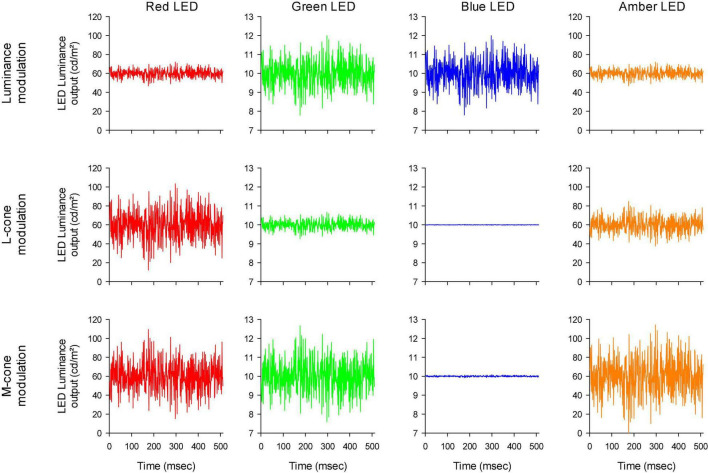
Luminance output of the four different LEDs as a function of time, to obtain luminance **(upper row)**, L-cone isolating **(middle row)** and M-cone isolating **(lower row)** TWN stimuli.

The stimulator contained four LED-based primaries (red, amber, green, and blue) with peak wavelengths at 646 nm (red), 598 nm (amber), 512 nm (green) and 466 nm (blue). The temporal profiles of the LED luminances are shown in Figure 2. Mean stimulus luminance was 140 cd/m^2^ (red: 60 cd/m^2^; amber: 60 cd/m^2^; green 10 cd/m^2^; blue: 10 cd/m^2^). The mean chromaticity was reddish. The temporal white noise (TWN) stimuli were presented in 512 ms epochs.

For the luminance stimuli, the four LEDs were modulated in phase and with constant relative ratios (Figure 2 upper row). Luminance contrast (defined as the Michelson contrast with the maximal and minimal luminance) was 22%. The human 10 degree cone (Stockman et al., 1993) and rod (V’_λ_; Wyszecki and Stiles, 1982) fundamentals of normal trichromats were used to calculate the L- and M-cone isolating stimuli according to the triple silent substitution paradigm (Kremers, 2003; Cao et al., 2015; Huchzermeyer and Kremers, 2016, 2017). In neither the L- nor the M-cone isolating stimuli, S-cone or rod excitation was modulated (i.e., the employed stimuli were silent substitutions for these photoreceptors). In the L-cone isolating stimuli, M-cones were also silenced and *vice versa*. The emission spectra of the LEDs were provided by the manufacturer and used for calculations of the sensitivity of each photoreceptor to each LED. Figure 2 second and third rows display the LED outputs for the L- and M-cone isolating stimuli, respectively (22% cone contrast in each condition; cone contrast was defined as the Michelson excitation contrast using the maximal and minimal cone excitation in the 512 ms episode). Observe that the outputs of the LEDs all had the same form and could only differ in magnitude (including mirror images of each other as, for instance, is the case for the red LED outputs in the L- and M-cone isolating conditions).

To validate the calculations, the photoreceptor excitations at each time during the stimulus were retrieved. This was done for each of the three stimulus conditions. Time dependent photoreceptor excitation *E*(*t*), quantified by cone or rod td, was:


(1) $$E_{i}\,\left(t\right)=P*K_{m}*\sum_{n}L_{n}\,\left(t\right)*C_{n}*S_{i,n}$$

Where *i* denotes the photoreceptor type (S-, M-, L-cones, or rods), *n* refers to the four LEDs (red, green, blue and amber); *P* is pupillary area (50.27 mm^2^ assuming an 8 mm diameter); *K*_*m*_ is the photopic luminous efficacy (683 lm/W); *L_*n*_(t)* is the luminance of the nth LED at time *t* in cd/m^2^ (shown in Figure 2); *C*_*n*_ is a conversion factor for the nth LED to convert the luminance in cd/m^2^ into W/sr.m^2^. *S_*i*,n_* is the sensitivity of photoreceptor *i* to the nth LED and is obtained by the integral over wavelength (λ) of the multiplication of the photoreceptor fundamental (*F_*i*_(*λ)) and the emission spectrum (*O_*n*_(*λ); given in W/sr.m^2^) of the nth LED:


(2) $$S_{i,n}=\int F_{i}\,\left(\lambda \right)*O_{n}\,{\left(\lambda \right)}^{*}\,d\,\lambda$$

**FIGURE 3:**
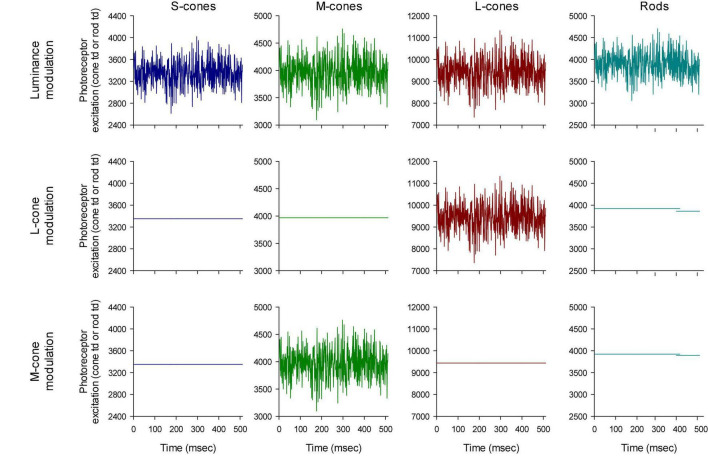
Validation of the stimulus conditions. The upper row shows that the four photoreceptor types modulate with equal temporal (white noise) profiles. The photoreceptor Michelson contrasts (defined as the difference between the maximal and the minimal excitation divided by twice the mean excitation) are equal for all photoreceptor types (22%). The middle row shows that only the L-cone excitation is modulated with a TWN profile in the L-cone isolating condition. The profile also equals the L-cone profile in the first row (luminance conditions) showing that the L-cone contrast is 22%. Similarly, the third row shows the M-cone temporal profile and the comparison with the profile in the luminance condition shows that the M-cone contrast is 22%.

**FIGURE 4:**
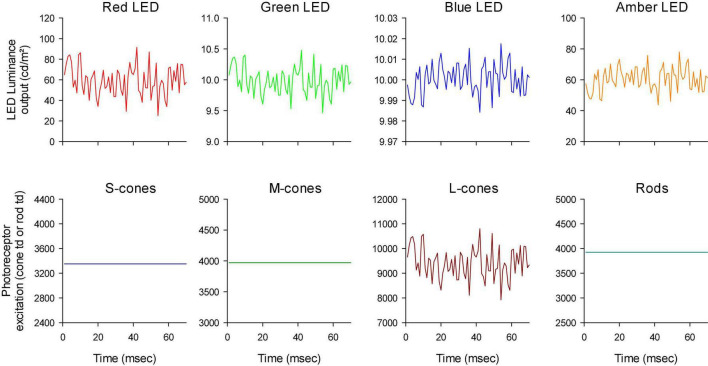
First 70 ms of the white noise stimuli. Furthermore, the outputs of the LEDs have different scaling to show that the luminance profiles were all similar although they could be mirror images of each other (compare e.g., the outputs of the red and blue LEDs).

The photoreceptor excitations *E*_*i*_(*t*) are displayed in Figure 3 as a function of time. An expansion of the first 70 ms is shown in Figure 4. The excitations in the three rows of Figure 3 show the excitation for the stimulus conditions displayed in the corresponding rows in Figure 2. They validate that the stimulus conditions were correctly calculated: In the luminance condition, all photoreceptors were modulated in phase and with equal contrast. In the L-cone isolating condition, L-cone contrast was identical to the L-cone contrast in the luminance condition (22%) while the excitations of the other photoreceptors were not modulated. Similarly, M-cone isolation resulted 22% M-cone contrast (identical to its contrast in the luminance condition) and 0% contrast in the other photoreceptor types. Furthermore, the mean cone excitations and thus the overall states of adaptation were identical in the three conditions. Note that this only validates our calculations. Mistakes and variability in the assumed fundamentals (for instance caused by genetic variation in photopigment absorption spectra or in preretinal absorption) may still be a cause of errors in the calculated photoreceptor excitations. The influence of variability in cone pigments, macular pigment and lens absorption have been calculated before (Huchzermeyer and Kremers, 2017). Assuming general variability in these parameters, the errors in calculated photoreceptor excitations are relatively minor.

To calculate rod td, we used the identical procedure as for cone td. As a result, rod td values are not identical with scotopic td values because the photopic (*K*_*m*_: 683 lm/W) instead of the scotopic luminous efficacy (*K’_*m*_*: 1,700 lm/W) was used. To obtain scotopic td, the rod td values should be multiplied by 2.498 (*K’_*m*_/K_*m*_*).

### Data acquisition and analysis

ERG responses to white noise stimuli (wnERGs) were recorded using an Espion E3 system (Diagnosys LLC, Lowell, MA, United States) in 512 ms sweeps. The signals were filtered DC to 300 Hz and digitized with 2,000 Hz sampling rate. Two recordings of 200 (luminance and L-cone isolating conditions) or 300 (M-cone isolating conditions) sweeps each were performed and the responses of the sweeps were averaged. The results of the two recordings were compared to estimate reproducibility of the recordings. In further analysis, the results of the two recordings were averaged. The data were analyzed using dedicated MATLAB programs and EXCEL worksheets.

The procedure of extracting IRFs from the wnERGs is described elsewhere (Zele et al., 2017). In brief, the stimulus and the wnERGs are cross-correlated by multiplying the response (*R*) and the stimulus (*S*) at every time stamp and by adding these multiplications. Then the stimulus was shifted in 1 ms steps relative and the procedure was repeated after each shift in time (τ). The IRF is given as a function of the shift in time as follows:


(3) $$I\,R\,F\,\left(\tau \right)=\frac{1}{N}\,\sum_{n=0}^{N}R\,\left(n\cdot \Delta \,t\right)\cdot S\,\left(n\cdot \Delta \,t-\tau \right)$$

Where *N* is the number of shifts and Δ*t* represents the 1 ms difference between the values at the different time stamps. For all calculations, the same stimulus functions were used, because the three stimulus conditions resulted in the same cone contrasts.

## Results

### Correlation between white noise stimuli

**FIGURE 5:**
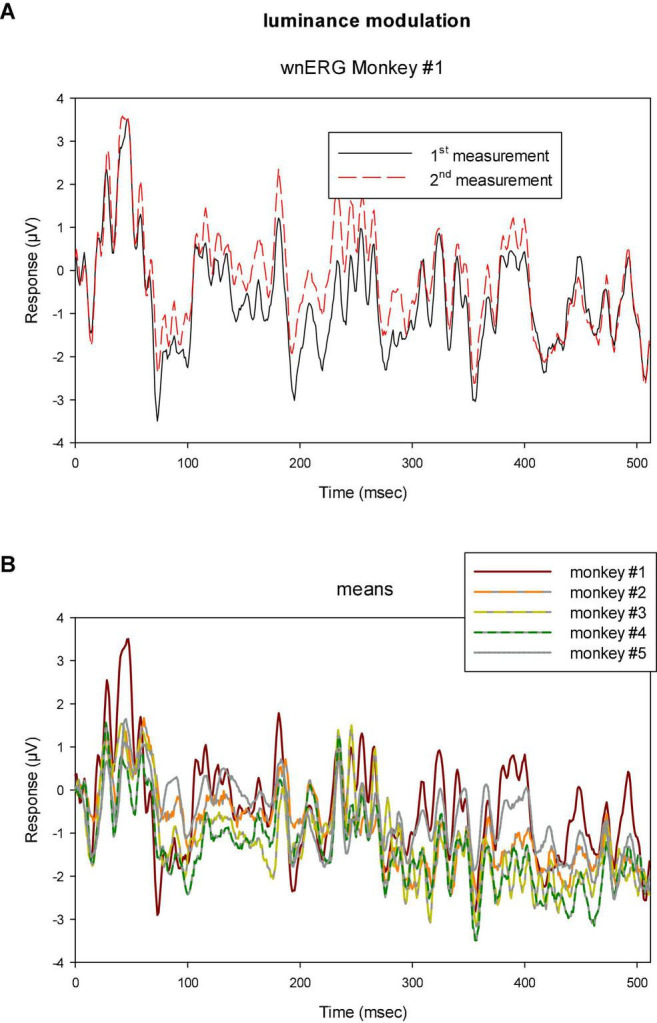
**(A)** Two sequential recordings with luminance TWN in monkey #1 showing that the responses were highly reproducible. **(B)** Means of two recordings to luminance TWN stimuli in five different monkeys. The responses show similar features for all animals although the differences are larger than those found between the two measurements within monkey #1.

Figure 5A shows an example of the two wnERGs measured in animal #1 to luminance TWN. The responses were corrected for drift differences during recordings following a previously described procedure (Kremers et al., 2021a). Briefly, the two measurements were given identical initial (at *t* = 0; mean of the values of the first 7 ms) and final (at *t* = 512 because the sweep duration was 512 ms; mean of the last 5 ms) values by addition or subtraction (Δ*_*i*_* and Δ*_*f*_*, respectively). The baseline drift between these values were assumed to be linear and relatively slow. Therefore, the correction (Δ*_*t*_*) for the measurement at time *t* was obtained by the linear interpolation between the two:$\Delta_{t}=\Delta_{i}+\frac{t}{512}\,\left(\Delta_{f}-\Delta_{i}\right)$. Correction for drift was particularly important when comparing two signals in time because drift may obscure a possible correlation.

Clearly, the responses resemble each other, indicating that the response can be reliably recorded. In addition, when the drift corrected mean responses to luminance TWN are plotted for all animals (Figure 5B), they display common features. The differences were, however, larger than those between the two measurements within a single animal showing inter-individual variability and differences in recording conditions.

**FIGURE 6:**
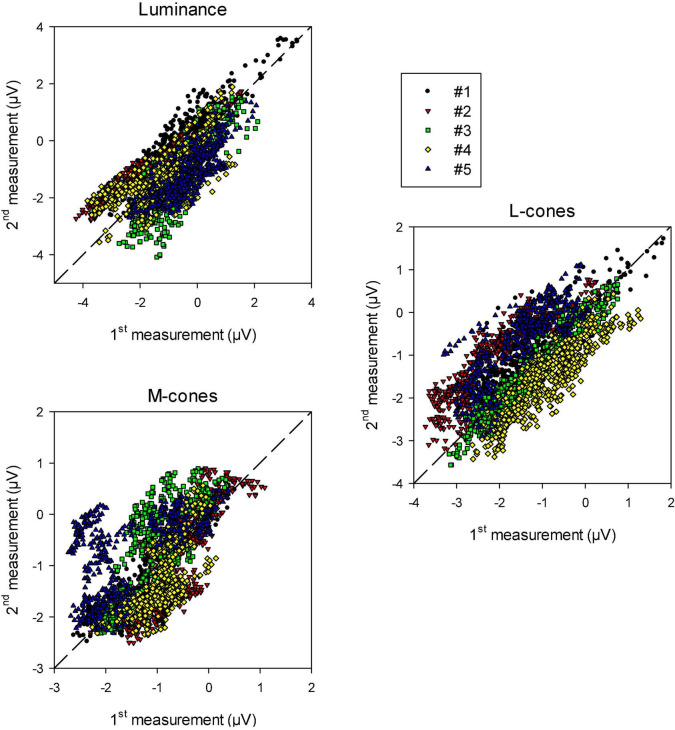
Recorded wnERG potentials in the 2nd recordings vs. those in the 1st recording at equivalent times relative to the white noise stimulus for luminance, L-cone isolating and M-cone isolating conditions. The results are displayed for the five different animals.

To quantify the reproducibility of the data, we plotted the (drift corrected) responses of the 2nd measurement as a function of those of the 1st measurement at the equivalent times during recording. The results are shown in Figure 6 for luminance, L-cone driven and M-cone driven wnERGs. In case the 1st and 2nd measurements were completely identical the data would lie on the (dashed) diagonal. It can be seen that there is a relationship between the responses obtained in 1st and 2nd measurements. The relationship was strongest for luminance stimuli and weakest for M-cone driven responses (even though the number of presentations were largest for the M-cone isolating stimuli; see section “Materials and methods”). In some animals (e.g., animal #5 in the two cone isolating conditions), the data showed distinct clusters. These clusters were probably caused by residual and relatively fast baseline shifts during the recordings that were not accounted for by the above described drift corrections.

We performed linear regressions on each data set. The squares of correlation coefficients (*r*^2^ values) give a quantification of how strongly the recordings resemble each other. They are given in Table 1 (upper row). The responses were correlated particularly for the luminance and L-cone driven wnERGs. M-cone driven responses were more variable (probably because the responses are smaller; see below).

**TABLE 1:** Squares of correlation coefficients for comparisons of responses obtained with the same stimulus conditions (5 comparisons each for 1st vs. 2nd measurements; 10 comparisons each for comparisons between different animals; all linear regressions were statistically significant).

Regression	Luminance	L-cone driven responses	M-cone driven responses
1st vs. 2nd measurement in same animal	0.72 (s.d.: 0.19; range: 0.44–0.94)	0.75 (s.d.: 0.10; range: 0.66–0.91)	0.54 (s.d.: 0.13; range: 0.36–0.69)
In different animals	0.53 (s.d.: 0.14; range: 0.32–0.74)	0.57 (s.d.: 0.21; range: 0.29–0.83)	0.74 (s.d.: 0.06; range: 0.67–0.85)

After averaging the 1st and 2nd (baseline corrected) measurement obtained in each animal, we plotted the response in one animal vs. the one in another animal. This was performed for the three stimulus conditions (data not shown; in total 10 plots for each condition). The squares of correlation coefficients are shown in the lower row of Table 1. Although M-cone driven responses between two measurements were intrinsically more variable in comparison with luminance and L-cone driven responses (because the responses were generally smaller and thus noisier as can be deduced from the different scaling of the axes in Figure 6; see also below), the mean square of the correlation coefficient was greater, indicating that the inter-individual variability was smaller. We verified the procedure by using two noise responses (i.e., recorded in the absence of a stimulus): no correlation was found. Furthermore, we considered the larger number of trials (300) per measurement to M-cone isolating stimuli compared to the luminance and L-cone isolating conditions where 200 trials per measurements were used. We increased the number of trials because we anticipated smaller M-cone driven responses and wanted to compensate for the lower SNR. We down-sampled offline the two measurements with M-cone isolating stimuli for monkey #1 to three measurements with 200 trials each and compared these measurements. We did not find a substantial difference (*r*^2^ with 300 trials was 0.61; *r*^2^ with 200 trials [three comparisons]: 0.58, 0.64, and 0.67). We are therefore confident that the larger number of trials in the M-cone driven responses did not influence the results.

We also obtained the square of correlation coefficients when plotting the results from the different conditions within the same animals against each other (five plots per comparison). The results are shown in Table 2 (upper row). Finally, we also correlated the responses in different animals and different stimulus conditions with each other (20 comparisons; Table 2 lower row). Correlations when comparing different stimulus conditions were similar when obtained in the same or in different animal, indicating that the inter-individual variability was less important than the response changes caused by a change in stimulus.

**TABLE 2:** Squares of correlation coefficients for comparisons of responses obtained with different stimulus conditions (5 comparisons each for 1st vs. 2nd measurements; 10 comparisons each for comparisons between different animals; all linear regressions were statistically significant).

Regression	Luminance vs. L-cone driven responses	Luminance vs. M-cone driven responses	L-cone vs. M-cone driven responses
In same animal	0.60 (s.d.: 0.13; range: 0.49–0.74)	0.46 (s.d.: 0.12; range: 0.31–0.63)	0.58 (s.d.: 0.13; range: 0.41–0.70)
In different animals	0.43 (s.d.: 0.16; range: 0.11–0.63)	0.44 (s.d.: 0.09; range: 0.28–0.62)	0.56 (s.d.: 0.10; range: 0.42–0.71)

### Distribution of white noise stimuli

**FIGURE 7:**
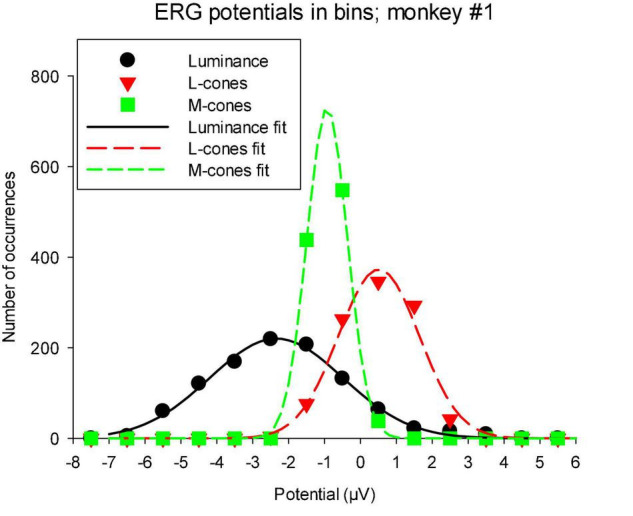
Distribution of wnERG potentials in monkey #1: the recordings were divided in bins of 1 μV width and the number of occurrences of wnERGs potentials within each bin is plotted as a function of the bin midpoint. The curves are fits of Gaussians to the data. The values of σ of the Gaussians are 1.84, 1.13, and 0.57 for luminance, L-cone driven and M-cone driven responses, respectively.

As mentioned in the “Materials and methods” section, the TWN stimulus delivers (quasi) random luminances or cone excitations with a Gaussian distribution. The distribution of measured ERG potentials can provide information about visual information processing in the ERG (Zele et al., 2017). We therefore determined the distribution of the macaque wnERG potentials (after correction for drifts). Figure 7 shows the results for animal #1, which are typical of all animals. It can be seen that the wnERG potentials also have distributions that can be described by Gaussians. Non-linearities like thresholding or saturation were expected to lead to substantial skewing of the distributions. This was not the case in the present recordings. The width of the fitted Gaussians (defined by σ of the fitted Gaussian) were largest for the luminance conditions (1.24 ± 0.37 μV; mean ± s.d.). The means and s.d.s of σ were 0.89 ± 0.21 and 0.74 ± 0.10 μV for L- and M-cone driven responses, respectively.

### Impulse response function

We obtained the IRFs by cross-correlating the TWN stimuli with the wnERGs (eq. 3). For these calculations, the wnERGs were not corrected for drift. However, we checked if a drift correction would affect the IRFs. The effect was found to be negligible.

**FIGURE 8:**
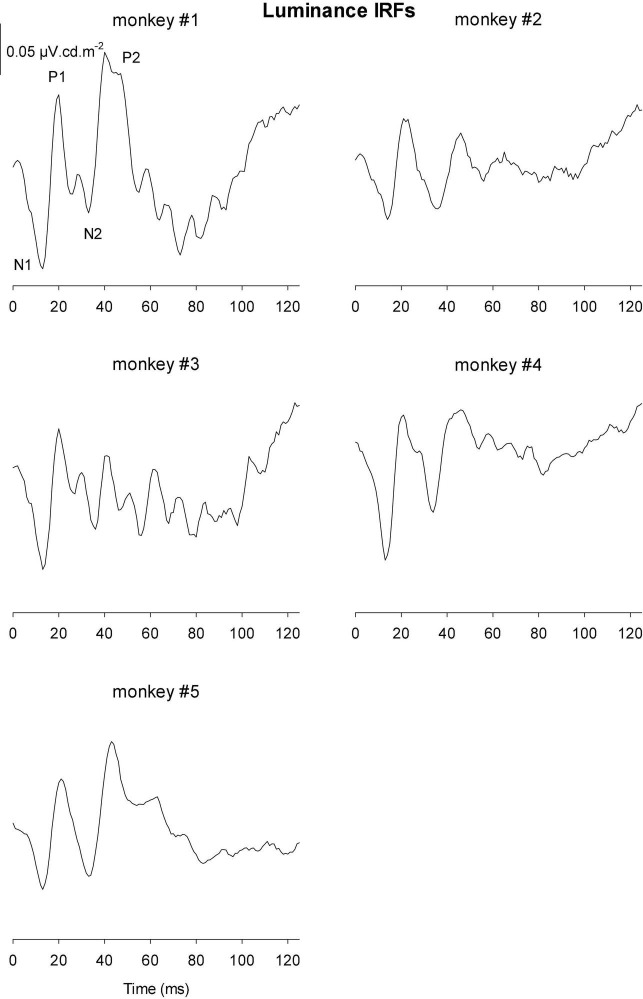
Derived impulse response functions (IRFs) from wnERGs to luminance TWN stimuli for five monkeys. In the IRF of monkey #1, the four components are defined.

The IRFs derived from luminance TWN measured in the five monkeys are shown in Figure 8. The luminance IRFs displayed an initial negativity (N1) with a latency of about 15 ms, resembling the a-wave in luminance flash ERGs. Furthermore, there was a secondary, b-wave-like, positivity with a latency of 20–25 ms. They were followed by a further negativity (N2) with delays between 35 and 40 ms and a positivity (P2) with a latency of 50–60 ms. In further contrast to the flash ERGs, no oscillatory potentials were present.

**FIGURE 9:**
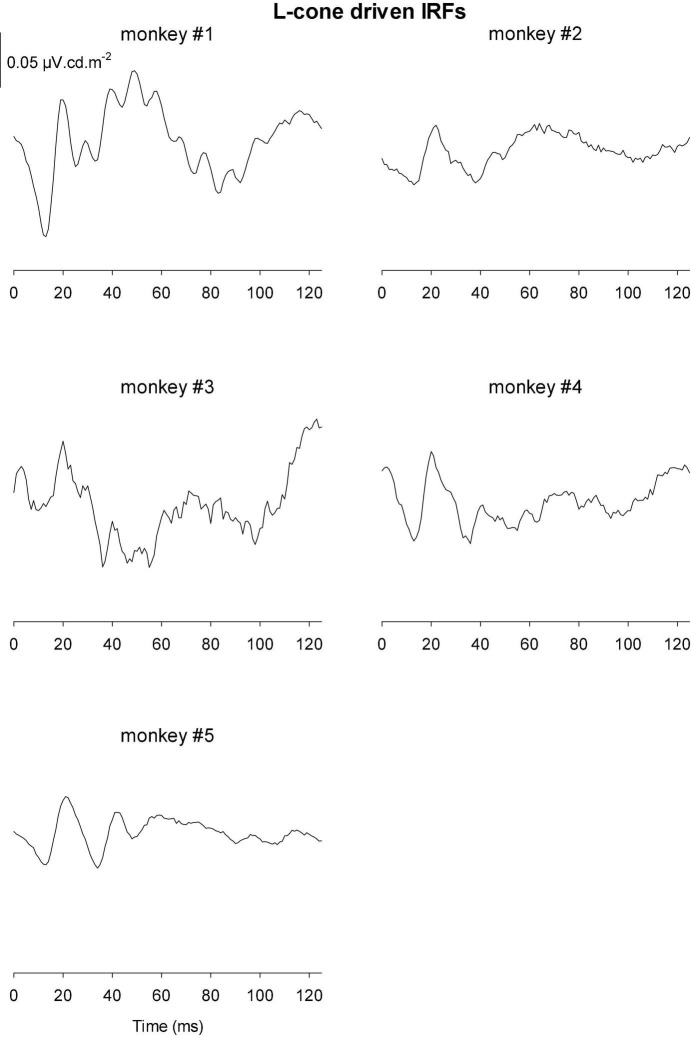
IRFs derived from L-cone driven wnERGs for five monkeys.

**FIGURE 10:**
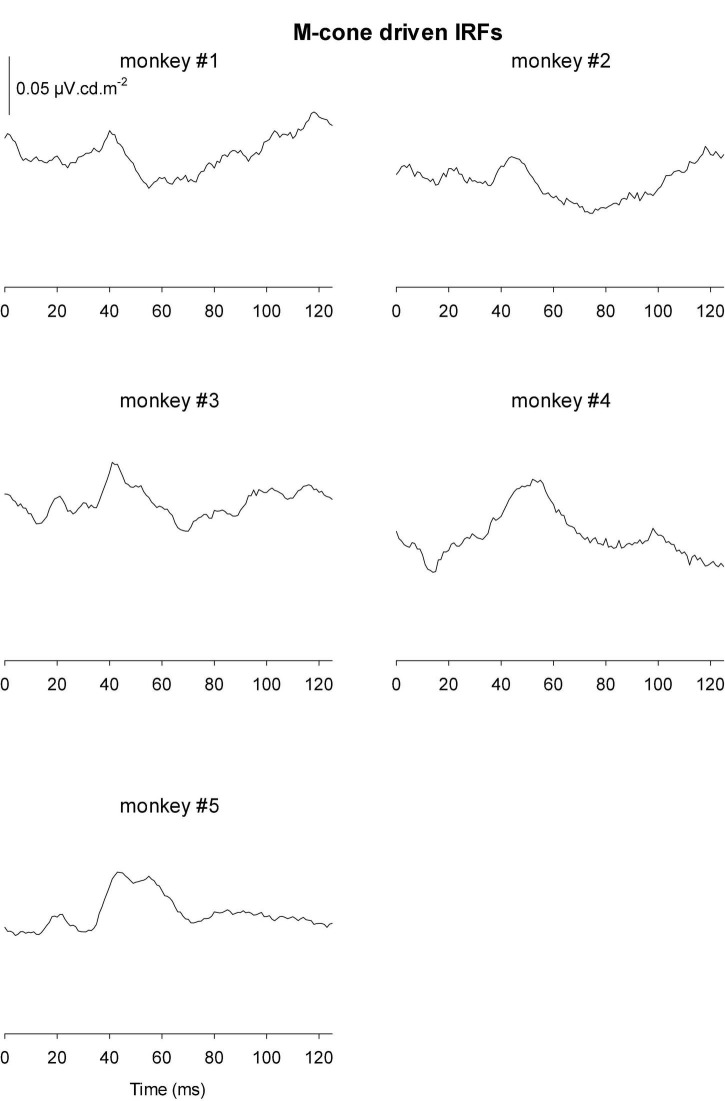
IRFs derived from M-cone driven wnERGs for five monkeys.

Figures 9, 10 Show the IRFs for L- and M-cone isolating conditions, respectively. The L-cone driven IRFs displayed similar components to the luminance IRFs, albeit with smaller amplitudes, probably because in the luminance conditions all photoreceptor types were stimulated. The P2 component could not always be clearly identified and their delay times could vary. The M-cone driven IRFs only displayed the P2 components and only hints of the earlier components.

**FIGURE 11:**
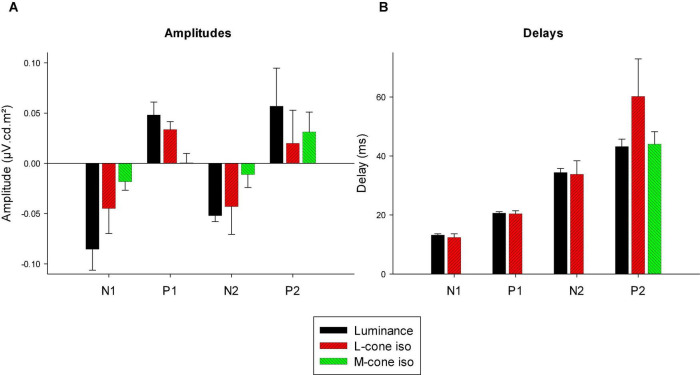
Amplitudes **(A)** and delays **(B)** of the four different components of luminance, L-cone driven and M-cone driven IRFs. Of the M-cone driven IRFs, only P2 was large enough to determine its delay.

We measured four parameters from the IRFs: The potentials in the first 5 ms were averaged to obtain a baseline measurement assuming that a clear response is absent in this period. The amplitudes of the four components were measured relative to this baseline. In addition, we registered the delays for these components. The mean (+ 1 s.d.) amplitudes are shown in Figure 11A. Clearly, the amplitudes were largest for the luminance stimuli. The amplitudes of the early M-cone driven IRF components (N1, P1, and N2) were smaller than those of L-cone driven IRFs despite the identical cone contrasts and were barely above noise (if present at all). From Figure 3, it can be seen that the mean L-cone excitation (in cone td) was larger than the mean M-cone excitation. However, both L- and M-cones were in a photopic state of adaptation where Weber’s law in expected to applicable, indicating that responses were expected to be equal for equal cone contrasts. Interestingly, the P2 components of M-cone driven IRFs were measurable and of similar amplitude as the L-cone driven P2 components.

Figure 11B displays the delays of different components. The early M-cone driven components were too small to give reliable delays. The early components of luminance and L-cone driven IRFs had very similar delays. The luminance and M-cone P2 components also had very similar delays. The L-cone driven P2 components, however, had larger delays and the variability of the data was also larger. This is in line with the earlier observation that the P2 components were not always easy to identify.

### Modulation transfer function

**FIGURE 12:**
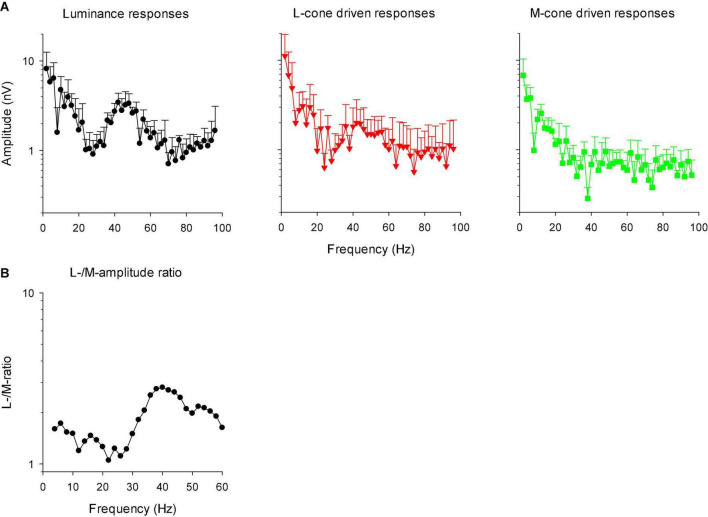
**(A)** Amplitude plots (mean + 1 SD) of the IRFs in the frequency domain after FFT (resulting in modulation transfer functions; MTFs). **(B)** The ratio of L- to M-cone driven response amplitudes as a function of temporal frequency. The data were smoothed by averaging the L-/M-ratio at the indicated frequency with those at the two adjacent frequencies.

In linear systems, the transformation of the IRF to the frequency domain results in the modulation transfer function (MTF) that is identical to the responses to sinusoidal stimuli of different temporal frequency. We therefore transformed the IRFs into MTFs through fast Fourier transform (FFT). Figure 12A shows the amplitude plots of the MTFs for responses to luminance, L-cone isolating and M-cone isolating stimuli. The MTFs showed two separate components: a low frequency component and a high frequency portion with a maximum at about 44 Hz. The two components were separated by a minimum at about 26 Hz. The high frequency component was absent in M-cone driven responses. We therefore propose that this component is the equivalent of the early deflections (N1 and P1) of the IRF in the time domain. The low frequency component was present for all three stimuli, indicating that this is the equivalent of P2 in the frequency domain. A time-frequency analysis of the signals may establish the connection between the IRF components and the frequency ranges in the MTFs more directly. From the L- and M-cone driven MTFs, we calculated L-/M-ratios as a function of temporal frequency. We smoothed the ratios by averaging the ratio at the concerning frequency with those obtained from the adjacent frequencies. The results are given as a function of temporal frequency in Figure 12B. The ratios were slightly larger than one at low frequencies and increased to values between 2 and 3 above about 30 Hz.

**FIGURE 13:**
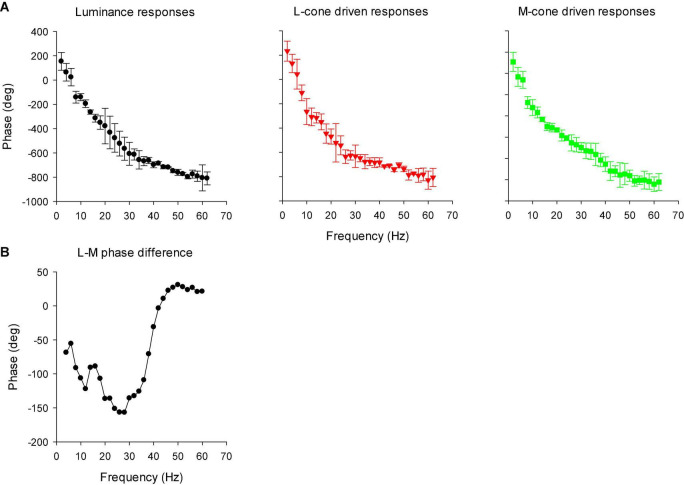
**(A)** Phase plots of the IRFs (mean ± 1 SD) in the frequency domain after FFT (resulting in modulation transfer functions; MTFs). **(B)** The difference between L- and M-cone driven response phases, as a function of temporal frequency. The data were smoothed by averaging the phase difference at the indicated frequency with those at the two adjacent frequencies.

Figure 13A displays the phase plots of the MTFs. The FFT returns phases between -180 and + 180°. We therefore adjusted phase by minimizing the phase differences (by adding or subtracting multiples of 360°) for the same stimulus conditions at adjacent frequencies and for different individual animals. Again, two frequency regions can be discerned. Below about 26 Hz, phase decreased strongly with increasing temporal frequency. At higher temporal frequencies the phase plots were shallower. At the transition (around 26 Hz), the standard deviations were relatively large. At these frequencies, amplitude was small and thus phase probably could not be reliably obtained. We performed separate linear regressions on the phase data between 10 and 22 Hz for the low frequency data and between 30 and 56 Hz for the high frequency data. For the low frequency portion, the slopes of the linear regressions were −23.7, −21.9, and −21.3°/Hz for the luminance, L-cone isolating and M-cone isolating conditions, respectively, equivalent to apparent latencies between 59.2 and 65.8 ms (Kommanapalli et al., 2014). The slopes of the linear regressions for the high frequency portions were −7.0, −5.6, and −13.1 deg/Hz for the luminance, L-cone driven responses and M-cone driven responses, respectively. This corresponds to apparent latencies of between 19.4, 15.5, and 36.4 ms for luminance, L- and M-cone driven responses, respectively.

We calculated the difference between the phases of L- and M-cone driven MTFs. The results are shown in Figure 13B (again after smoothing the data by averaging the value at the concerning frequency with those at adjacent frequencies). The phase differences were large at low frequencies and close to zero at frequencies above 40 Hz.

## Discussion

In the present study, we measured ERG responses (wnERGs) to stimuli with white noise temporal profiles. We combined the stimulation with the silent substitution technique to isolate the responses of single photoreceptor types. With the silent substitution technique, stimulus strength (in terms of cone contrast) or mean excitation (in terms of cone td) are invariants so that their influence on the ERG can be independently studied. Theoretically, any combination of photoreceptor stimulation can be chosen although, in practice, this is limited by the gamut of the stimulator. Photoreceptor isolation is thus a special combination where the stimulation in all but one photoreceptor type is zero. In the current study we measured the responses to luminance (i.e., all photoreceptors in phase and with equal contrast) and to L- and M-cone isolating stimuli. The L- and M-cone contrasts in the isolating conditions were equal and also equal to their contrasts in the luminance conditions. Furthermore, mean luminance and chromaticity, and thus the state of adaptation, were equal in the three conditions. As a consequence, the data obtained from these conditions can be directly compared. It would be interesting to also record ERGs to isoluminant chromatic stimuli. However, isoluminance most probably differs between different individuals (mainly due to inter-individual variability in the ratio between L- and M-cone numbers) and thus would need an individual stimulus calibration of isoluminance.

In the current study, the wnERGs were analyzed in several ways to characterize the generating mechanisms. First, the different responses were correlated with each other to obtain information about similarities and differences in their retinal origins. Second, the distribution of measured ERG potentials was analyzed. Third, the responses were used to derive IRFs in the time domain. These can be compared with those obtained in human subjects (Saul and Still, 2016; Zele et al., 2017). The IRFs are linear approximations of flash ERGs. Differences with flash ERGs therefore may give an indication about non-linear mechanisms involved in the generation of the flash ERG. Finally, the modulation transfer functions (MTFs) in the frequency domain can be compared with each other and with responses to sine-wave stimuli of different temporal frequencies (Viswanathan et al., 2002; Pangeni et al., 2010; Kremers and Pangeni, 2012; Kommanapalli et al., 2014). The wnERGs therefore can be an efficient method to characterize the ERG generating mechanisms. The stimuli may be considered to be more natural and more physiological than flashes or other repetitive waveforms. The stimulus strengths are moderate so that some non-linearities, such as saturation, may be weaker.

### Comparison of L- and M-cone driven responses

We found substantial differences between the ERGs elicited by L- and M-cone isolating stimuli. The wnERGs for luminance and L-cone isolating conditions showed the strongest correlation in repeated measurements, indicating that these responses are most robust and reproducible. Their ERGs had broader distributions of their potential and thus differed more strongly from a distribution that would be solely determined by internal noise. M-cone driven responses were smaller, were more variable in different measurements and had narrower distributions. However, they were still robust. Interestingly, the correlations of responses obtained from different animals were strongest for the M-cone isolating conditions. This indicates that inter-individual variability is smaller with M-cone isolating stimuli than with the other two conditions. We propose that M-cone driven responses mainly originate in the L-/M-opponent retinal pathway that projects to the parvocellular layers of the lateral geniculate nucleus (LGN). Previous data indicate that this pathway shows less inter-individual variability than the luminance-sensitive pathway that projects to the magnocellular LGN layers (Kremers et al., 2000, 2021a). Significant M-cone inputs to the L-/M-cone opponent channel have also been identified in ERG recordings (Kremers and Pangeni, 2012; Kommanapalli et al., 2014) in psychophysical measurements (Parry et al., 2016) in pupillometric data (Murray et al., 2018; Woelders et al., 2018; Parry et al., 2020) and in VEP recordings (Barboni et al., 2017) obtained with human subjects. The luminance pathway is L-cone dominated in most trichromats (Kremers and Pangeni, 2012; Kommanapalli et al., 2014; Jacob et al., 2015; Kremers et al., 2021a). This is a possible explanation why luminance and L-cone driven responses were more variable in different animals despite their robustness within an individual. Furthermore, it may explain why luminance and L-cone driven responses within the same individuals were more strongly correlated (*r*^2^ = 0.60) than when M-cone driven responses were compared with luminance responses (*r*^2^ = 0.46; see “Results”).

Compared to L-cone driven responses, the early IRF components (N1 and P1) and their putative equivalents in the MTFs (at frequencies between 30 and 60 Hz) of responses to M-cone isolating stimuli were substantially smaller although equal cone contrasts were employed in the two stimuli. As a result, the L-/M-ratios were substantially larger than unity (see e.g., Figure 12B). Large L-/M-cone driven ERGs ratios were also found in the responses to high frequency sine wave stimuli in macaques (Kremers et al., 2021a) and in human subjects (Kremers and Pangeni, 2012; Parry et al., 2012; Kommanapalli et al., 2014). In addition, it was found in human observers that L-/M-ratios in high frequency ERGs correlate with those obtained from psychophysical measurements that tap the luminance pathway (Brainard et al., 2000; Kremers et al., 2000, 2003), with L- vs. M-cone numbers (Brainard et al., 2000) and with L- vs. M-pigment content (Kremers et al., 2000). The phase differences of the L- and M-cone driven MTFs at the characteristic frequencies of the N1 and P1 components (30 Hz and higher) were small (Figure 13B). Overall, these results suggest that the N1 and P1 components reflect activity of the luminance sensitive magnocellular pathway and that, as in human subjects, the macaque retina is L-cone dominated.

The P2 component had similar amplitudes for L- and M-cone isolating conditions (Figure 11) and also in the frequency domain the L-/M-ratio was close to unity at the frequencies that define P2 (below about 20 Hz; Figure 12B). At these frequencies, the L-M phase difference was large (Figure 13B), indicating substantial cone opponency. These results therefore suggest that the P2 component originates in activity of the parvocellular L-M opponent pathway.

### Luminance impulse response functions and their comparison to flash electroretinograms

IRFs were obtained from the cross-correlation of the TWN stimulus and the recorded wnERG. This procedure has been used in other areas of vision research mainly such as single cell recordings (e.g., Field et al., 2010). The procedure is akin to the procedure of extracting local responses from the multifocal stimulation (Sutter and Tran, 1992). It has only recently been used in ERG measurements (Saul and Still, 2016; Zele et al., 2017; Adhikari et al., 2019; Wang et al., 2019). It was found that using natural white noise (where, in contrast to the Gaussian white noise stimuli used in the current study, the luminance at a certain time depends on the luminance in the previous instant) gave slightly larger IRFs (Saul and Still, 2016). This finding can possibly be explained because natural white noise stimuli probably have larger amplitudes at lower temporal frequencies whereas the Gaussian white noise has equal amplitudes at all frequencies. Since substantial ERG responses can only be found at frequencies below about 100 Hz and are overall low-pass, it can be expected that responses increase when lower frequencies are represented in the stimulus. We have performed ERG recordings in mice using TWN stimuli. We obtained substantially larger IRFs when the stimulus did not contain frequencies above 20 Hz, and that resembles the natural white noise stimuli Stallwitz (unpublished observations).

IRFs are expected to be identical to the responses to flashes if the underlying mechanism is linear. The comparison between the IRFs and flash ERGs may therefore give information on the differences in underlying mechanisms and on non-linearities that are involved in the flash ERGs.

**FIGURE 14:**
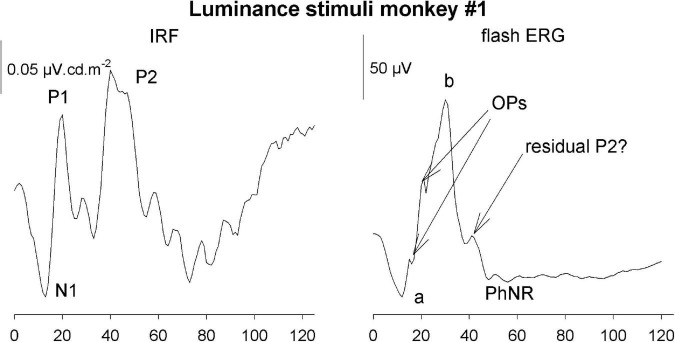
Comparison of the luminance IRF from monkey #1 with the response to a 5.68 cd.s/m^2^ flash upon a 38 cd/m^2^ background.

The IRFs in the current study were obtained from measurements in photopic conditions. They therefore resemble photopic flash ERGs with an initial a-wave-like negativity followed by a b-wave-like positivity, indicating that the IRF components and their equivalents in the flash ERG are homologs and have the same cellular origins (Frishman, 2006; see Figure 14).

However, there are also differences. One clear difference is the absence of oscillatory potentials (OPs) in the IRFs. This was also observed in IRFs obtained from wnERGs in human subjects (Saul and Still, 2016; Zele et al., 2017). Furthermore, a photopic negative response (PhNR) was not detectable, Flashes are typically stimuli with high contrasts where the luminance or cone excitation during the flash can be orders of magnitude larger than those of the background. Assuming that the duration of the flash used in the recording shown in Figure 14 was 5 ms (longest flash duration recommended by the ISCEV standards), the flash luminance was 1,136 cd/m^2^ or more. With a 38 cd/m^2^ background this results a Weber contrast of at least 3,000% (1,136/38*100%). This may activate non-linearities that are not present in the IRFs. The OPs and the PhNR therefore may originate in non-linear response mechanisms.

On the other hand, the P2 component that we identified in the IRFs are generally not observed in the flash ERGs. We argue that P2 may originate in activity of the chromatic sensitive parvocellular retinal pathway. Flash ERGs generally contain a strong luminance component and therefore may not elicit a P2-like response component. The small peak on the descending limb in Figure 14 possibly is a residual P2 response.

## Conclusion

wnERG recordings can be combined with silent substitution thereby providing an efficient way to characterize mechanisms that lead to an ERG response. They provide information about reproducibility and inter-individual variability in ERG responses. The comparison of IRFs with flash ERGs may help to determine involved cell types and may help to identify non-linearities. Distribution of potentials may provide additional information about involved non-linearities. IRFs in the time domain and their pendants in the frequency domain (MTFs) may give information about the photoreceptoral inputs and involved retino-geniculate pathways.

## Data availability statement

The raw data supporting the conclusions of this article will be made available by the authors, without undue reservation.

## Ethics statement

The animal study was reviewed and approved by the Institutional Animal Care and Use Committee of the University of Houston (#16-046).

## Author contributions

JK: conception and design of the study, construction of the stimuli, planning and performance of the experiments, data analysis, and writing and editing of the manuscript. AA: performance of the experiments, data analysis, and editing of the manuscript. NRAP: construction of the stimuli and editing of the manuscript. NBP: performance of the experiments. LF: planning and performance of the experiments, and editing of the manuscript. All authors contributed to the article and approved the submitted version.

## References

[B1] Adhikari P. Zele A. J. Cao D. Kremers J. Feigl B. (2019). The melanopsin-directed white noise electroretinogram (wnERG). *Vis. Res.* 164 83–93. 10.1016/j.visres.2019.08.007 31542209

[B2] Barboni M. T. S. Nagy B. V. Martins C. M. G. Bonci D. M. O. Hauzman E. Aher A. (2017). L-/M-cone opponency in visual evoked potentials of human cortex. *J. Vis.* 17:20. 10.1167/17.9.20 28837966

[B3] Brainard D. H. Roorda A. Yamauchi Y. Calderone J. B. Metha A. B. Neitz M. (2000). Functional consequences of the relative numbers of L and M cones. *J. Optical Soc. Am. A* 17 607–614. 10.1364/josaa.17.000607 10708042

[B4] Cao D. Nicandro N. Barrionuevo P. A. (2015). A five-primary photostimulator suitable for studying intrinsically photosensitive retinal ganglion cell functions in humans. *J. Vis.* 15:27. 10.1167/15.1.27 PMC4528566 25624466

[B5] Challa N. K. McKeefry D. Parry N. R. A. Kremers J. Murray I. J. Panorgias A. (2010). L- and M-cone input to 12Hz and 30Hz flicker ERGs across the human retina. *Ophthalmic Physiol. Optics* 30 503–510. 10.1111/j.1475-1313.2010.00758.x 20883333

[B6] Dawson W. W. Trick G. L. Litzkow C. A. (1979). Improved electrode for electroretinography. *Invest Ophthalmol. Vis. Sci.* 18 988–991. 478786

[B7] Field G. D. Gauthier J. L. Sher A. Greschner M. Machado T. A. Jepson L. H. (2010). Functional connectivity in the retina at the resolution of photoreceptors. *Nature* 467 673–677. 10.1038/nature09424 20930838 PMC2953734

[B8] Frishman L. J. (2006). “Origins of the electroretinogram,” in *Principles and Practice of Clinical Electrophysiology of Vision*, eds Heckenlively J. R. Arden G. B. (London: The MIT Press), 139–183.

[B9] Hood D. C. (2000). Assessing retinal function with the mulifocal technique. *Prog. Retinal Eye Res.* 19 607–646. 10.1016/s1350-9462(00)00013-6 10925245

[B10] Huchzermeyer C. Kremers J. (2016). Perifoveal L- and M-cone-driven temporal contrast sensitivities at different retinal illuminances. *J. Opt. Soc. Am. Opt. Image Sci. Vis.* 33 1989–1998. 10.1364/JOSAA.33.001989 27828102

[B11] Huchzermeyer C. Kremers J. (2017). Perifoveal S-cone and rod-driven temporal contrast sensitivities at different retinal illuminances. *J. Opt. Soc. Am. Opt. Image Sci. Vis.* 34 171–183. 10.1364/JOSAA.34.000171 28157843

[B12] Jacob M. M. Pangeni G. Gomes B. D. Souza G. S. Da Silva Filho M. Silveira L. C. L. (2015). The spatial properties of L- and M-cone inputs to electroretinograms that reflect different types of post-receptoral processing. *PLoS One* 10:e0121218. 10.1371/journal.pone.0121218 25785459 PMC4364754

[B13] Kommanapalli D. Murray I. J. Kremers J. Parry N. R. McKeefry D. J. (2014). Temporal characteristics of L- and M-cone isolating steady-state electroretinograms. *J. Opt. Soc. Am. Opt. Image Sci. Vis.* 31 A113–120. 10.1364/JOSAA.31.00A113 24695158

[B14] Kremers J. (2003). The assessment of L- and M-cone specific electroretinographical signals in the normal and abnormal retina. *Prog. Retinal Eye Res.* 22 579–605. 10.1016/s1350-9462(03)00049-1 12892643

[B15] Kremers J. Aher A. J. Parry N. R. A. Patel N. B. Frishman L. J. (2021a). Comparison of macaque and human L- and M-cone driven electroretinograms. *Exp. Eye Res.* 206:108556. 10.1016/j.exer.2021.108556 33794198 PMC8111691

[B16] Kremers J. Aher A. J. Popov Y. Mirsalehi M. Huchzermeyer C. (2021b). The influence of temporal frequency and stimulus size on the relative contribution of luminance and L-/M-cone opponent mechanisms in heterochromatic flicker ERGs. *Doc. Ophthalmol.* 143 207–220. 10.1007/s10633-021-09837-9 33886039 PMC8494685

[B17] Kremers J. Link B. (2008). Electroretinographic responses that may reflect activity of parvo- and magnocellular post-receptoral visual pathways. *J. Vis.* 8 1–14. 10.1167/8.15.11 19146295

[B18] Kremers J. Pangeni G. (2012). Electroretinographic responses to photoreceptor specific sine wave modulation. *J. Optic. Soc. Am. A* 29 A309–316. 10.1364/JOSAA.29.00A306 22330394

[B19] Kremers J. McKeefry D. J. Murray I. J. Parry N. R. A. (2020). Developments in non-invasive visual electrophysiology. *Vis. Res.* 174 50–56. 10.1016/j.visres.2020.05.003 32540518

[B20] Kremers J. Pangeni G. Tsaousis K. T. McKeefry D. Murray I. J. Parry N. R. (2014). Incremental and decremental L- and M-cone driven ERG responses: II. Sawtooth stimulation. *J. Opt. Soc. Am. Opt. Image Sci. Vis.* 31 A170–178. 10.1364/JOSAA.31.00A17 24695166

[B21] Kremers J. Rodrigues A. R. Silveira L. C. L. da Silva-Filho M. (2010). Flicker ERGs representing chromaticity and luminance signals. *Investig. Ophthalmol. Vis. Sci.* 51 577–587. 10.1167/iovs.09-3899 19737882

[B22] Kremers J. Scholl H. P. N. Knau H. Berendschot T. T. J. M. Usui T. Sharpe L. T. (2000). L/M cone ratios in human trichromats assesed by psychophysics, electroretinograpy, and retinal densitometry. *J. Opt. Soc. Am.* 17 517–526. 10.1364/josaa.17.000517 10708033

[B23] Kremers J. Stepien M. W. Scholl H. P. N. Saito C. A. (2003). Cone selective adaptation influences L- and M-cone driven signals in electroretinography and psychophysics. *J. Vis.* 3 146–160. 10.1167/3.2.3 12678617

[B24] Luo X. Patel N. B. Rajagopalan L. P. Harwerth R. S. Frishman L. J. (2014). Relation between macular retinal ganglion cell/inner plexiform layer thickness and multifocal electroretinogram measures in experimental glaucoma. *Invest. Ophthalmol. Vis. Sci.* 55 4512–4524. 10.1167/iovs.14-13937 24970256 PMC4106250

[B25] Maguire J. Parry N. R. Kremers J. Kommanapalli D. Murray I. J. McKeefry D. J. (2016). Rod electroretinograms elicited by silent substitution stimuli from the light-adapted human eye. *Transl. Vis. Sci. Technol.* 5:13. 10.1167/tvst.5.4.13 27617180 PMC5015991

[B26] Marmarelis P. Z. Marmarelis V. Z. (1978). *Analysis of Physiological Systems.* New York, NY: Plenum Press.

[B27] Marmarelis P. Z. Naka K. I. (1973). Nonlinear analysis and synthesis of receptive-field responses in the catfish retina. 3. Two-input white-noise analysis. *J. Neurophysiol.* 36 634–648. 10.1152/jn.1973.36.4.634 4713312

[B28] McKeefry D. Kremers J. Kommanapalli D. Challa N. K. Murray I. J. Maguire J. (2014). Incremental and decremental L- and M-cone-driven ERG responses: I. Square-wave pulse stimulation. *J. Opt. Soc. Am. Opt. Image Sci. Vis.* 31 A159–169. 10.1364/JOSAA.31.00A159 24695165

[B29] Murray I. J. Kremers J. McKeefry D. Parry N. R. A. (2018). Paradoxical pupil responses to isolated M-cone increments. *J. Opt. Soc. Am. Opt. Image Sci. Vis.* 35 B66–71. 10.1364/JOSAA.35.000B66 29603924

[B30] Pangeni G. Horn F. K. Kremers J. (2010). A new interpretation of components in the ERG signals to sine wave luminance stimuli at different temporal frequencies and contrasts. *Vis. Neurosci.* 27 79–90. 10.1017/S0952523810000179 20796325

[B31] Parry N. R. A. McKeefry D. J. Kremers J. Murray I. J. (2016). A dim view of M-cone onsets. *J. Optic. Soc. Am. A* 33 A207–213. 10.1364/JOSAA.33.00A207 26974925

[B32] Parry N. R. A. Rodrigo-Diaz E. Murray I. J. (2020). Anomalous pupillary responses to M-cone onsets are linked to L:M ratio. *J. Opt. Soc. Am. Opt. Image Sci. Vis.* 37 A163–169. 10.1364/JOSAA.382262 32400539

[B33] Parry N. R. Murray I. J. Panorgias A. McKeefry D. J. Lee B. B. Kremers J. (2012). Simultaneous chromatic and luminance human electroretinogram responses. *J. Physiol.* 590 3141–3154. 10.1113/jphysiol.2011.226951 22586211 PMC3406396

[B34] Rangaswamy N. V. Shirato S. Kaneko M. Digby B. I. Robson J. G. Frishman L. J. (2007). Effects of spectral characteristics of ganzfeld stimuli on the photopic negative response (PhNR) of the ERG. *Invest. Ophthalmol. Vis. Sci.* 48 4818–4828. 10.1167/iovs.07-0218 17898309 PMC2100398

[B35] Robson A. G. Frishman L. J. Grigg J. Hamilton R. Jeffrey B. G. Kondo M. (2022). ISCEV Standard for full-field clinical electroretinography (2022 update). *Doc. Ophthalmol*. 144 165–177. 10.1007/s10633-022-09872-0 35511377 PMC9192408

[B36] Saul A. B. Still A. E. (2016). Multifocal electroretinography in the presence of temporal and spatial correlations and eye movements. *Vision* 1:1010003. 10.3390/vision1010003 31740628 PMC6849053

[B37] Stallwitz N. Joachimsthaler A. Kremers J. (unpublished). *Luminance White Noise ERGs in Mice*. 10.3389/fnins.2023.1211329 PMC10423813 37583414

[B38] Stockman A. MacLeod D. I. A. Johnson N. E. (1993). Spectral sensitivities of the human cones. *J. Optic. Soc. Am. A* 10 2491–2521. 10.1364/josaa.10.002491 8301403

[B39] Sutter E. E. Tran D. (1992). The field topography of ERG components in man-I. The photopic luminance response. *Vis. Res.* 32 433–446. 10.1016/0042-6989(92)90235-b 1604830

[B40] Tsai T. I. Jacob M. M. McKeefry D. Murray I. J. Parry N. R. A. Kremers J. (2016). Spatial properties of L- and M-cone driven incremental (On-) and decremental (Off-) electroretinograms: evidence for the involvement of multiple post-receptoral mechanisms. *J. Optic. Soc. Am. A* 33 A1–11. 10.1364/JOSAA.33.0000A1 26974913

[B41] Viswanathan S. Frishman L. J. Robson J. G. (2002). Inner-retinal contributions to the photopic sinusoidal flicker electroretinogram of macaques. *Documenta Ophthalmol.* 105 223–242. 10.1023/a:1020505104334 12462445

[B42] Wang J. Saul A. Smith S. B. (2019). Activation of sigma 1 receptor extends survival of cones and improves visual acuity in a murine model of retinitis pigmentosa. *Invest. Ophthalmol. Vis. Sci.* 60 4397–4407. 10.1167/iovs.19-27709 31639826 PMC6808049

[B43] Woelders T. Leenheers T. Gordijn M. C. M. Hut R. A. Beersma D. G. M. Wams E. J. (2018). Melanopsin- and L-cone-induced pupil constriction is inhibited by S- and M-cones in humans. *Proc. Natl. Acad. Sci. U S A.* 115 792–797. 10.1073/pnas.1716281115 29311335 PMC5789936

[B44] Wyszecki G. Stiles W. (1982). *Color Science: Concepts and Methods, Quantitative Data and Formulas.* New York, NY: John Wiley & Sons.

[B45] Zele A. J. Feigl B. Kambhampati P. K. Aher A. McKeefry D. Parry N. (2017). A temporal white noise analysis for extracting the impulse response function of the human electroretinogram. *Transl. Vis. Sci. Technol.* 6:1. 10.1167/tvst.6.6.1 29109907 PMC5666911

